# Collagen patch cover facilitates recovery of bowel function after laparoscopic colectomy

**DOI:** 10.1186/s12893-024-02339-w

**Published:** 2024-02-20

**Authors:** Pin-Yang Huang, Meng-Che Tsai, Kee-Thai Kiu, Min-Hsuan Yen, Tung-Cheng Chang

**Affiliations:** 1 Department of General Medicine, Taipei Medical University Shuang-Ho Hospital, No. 291, Zhongzheng Road, Zhonghe District, New Taipei City, 235 Taiwan; 2 Department of General Medicine, Chiayi Chang Gung Memorial Hospital, Chiayi County, No. 291, Zhongzheng Road, Zhonghe District, New Taipei City, 235 Taiwan; 3 Division of Colorectal Surgery, Department of Surgery, Taipei Medical University Shuang-Ho Hospital, No. 291, Zhongzheng Road, Zhonghe District, New Taipei City, 235 Taiwan; 4 Division of Colorectal Surgery, Department of Surgery, School of Medicine, College of Medicine, Taipei Medical University, No. 291, Zhongzheng Road, Zhonghe District, New Taipei City, 235 Taiwan

**Keywords:** Collagen patch cover, Bowel recovery, Laparoscopic colectomy

## Abstract

**Background:**

Numerous factors can influence bowel movement recovery and anastomotic healing in colorectal surgery, and poor healing can lead to severe complications and increased medical expenses. Collagen patch cover (CPC) is a promising biomaterial that has been demonstrated to be safe in animal models and has been successfully applied in various surgical procedures in humans. This study.

**Methods:**

A retrospective review of medical records from July 2020 to June 2022 was conducted to identify consecutive patients who underwent laparoscopic colectomy. Patients who received CPC at the anastomotic site were assigned to the collagen group, whereas those who did not receive CPC were assigned to the control group.

**Results:**

Data from 241 patients (collagen group, 109; control group, 132) were analyzed. Relative to the control group, the collagen group exhibited a faster recovery of bowel function, including an earlier onset of first flatus (2.93 days vs. 3.43 days, *p* < 0.01), first defecation (3.73 days vs. 4.18 days, *p* = 0.01), and oral intake (4.30 days vs. 4.68 days, *p* = 0.04). CPC use was also associated with lower use of postoperative intravenous analgesics. The complication rates in the two groups did not differ significantly.

**Conclusions:**

CPCs can be safely and easily applied to the anastomotic site during laparoscopic colectomy, and can accelerate bowel movement recovery. Further studies on the effectiveness of CPCs in colorectal surgery involving larger sample sizes are required.

**Clinical trial registration:**

ClinicalTrials.gov registration number: NCT05831956 (26/04/2023).

## Introduction

Wound healing occurs in four distinct phases: hemostasis, inflammation, proliferation, and tissue remodeling. Numerous local and systemic factors influence these phases [1]. Local factors include infection, presence of foreign bodies, inadequate oxygenation, and other issues that affect a wound, whereas systemic factors may involve age, sex, ischemia, stress, medication use, obesity, cigarette or alcohol use, nutritional status, and immunocompromised status [1].

Bowel recovery and skin recovery differ in several aspects. Firstly, they involve distinct types of collagen and collagenase activity for wound healing. High collagenase activity can result in collagen dissolution, leading to lower strength at the anastomotic site [2]. Additionally, factors such as shear stress, the presence of bacteria that can impact anastomotic healing, and variations in vascular perfusion are more sufficient in the intestinal environment. These differences resulted in the superior healing outcomes in bowel recovery compared to skin recovery [2]. The cellular and histological processes involved in colonic healing are well understood, however the physiological process involved in such healing requires further clarification [3, 4]. Unlike the straightforward observation of skin healing, the assessment of colonic resection and anastomotic healing requires indirect monitoring of specific parameters. These include the evaluation of parameters such as the color and volume of ascites from the drainage tube, time to the first flatus and defecation, as well as monitoring patient-reported symptoms. These indicators were employed to assess the condition of bowel recovery in patients. Complications such as anastomotic leakage, bleeding, or stricture lead to a longer hospital stay and increased mortality and morbidity [5]. Among these complications, anastomotic leakage is the most severe. Various strategies have been developed for reducing the rate of anastomotic leakage associated with colorectal surgery, including colon preparation or antibiotic administration. However, reducing the incidence of anastomotic leakage remains a great challenge for surgeons [6].

Besides local and systemic factors that influence healing, the use of various biomaterials, including collagen, plays a crucial role in all four phases of wound healing, promoting the overall process [7]. Collagen patch covers (CPCs) derived from bovine, equine, avian, or porcine sources have been applied to various wound sites to promote healing [8]. Previous studies have reported that CPC use results in improved outcomes; however, these studies were in vitro or animal-based studies [9–13]. While various studies have shown the safety and benefits of applying CPC to anastomotic sites in colorectal and gastric surgical procedures in humans [14, 15], these investigations have typically involved a limited number of patients. Consequently, there is a need for extensive research on the utilization of CPC in anastomoses after colorectal resection, particularly on a larger sample size. To investigate the clinical outcomes of CPC use for human colonic anastomosis, a retrospective case–control study was conducted and the effects of CPC use in elective laparoscopic colectomy surgery were assessed.

## Materials and methods

### Patients selection

**Fig. 1:**
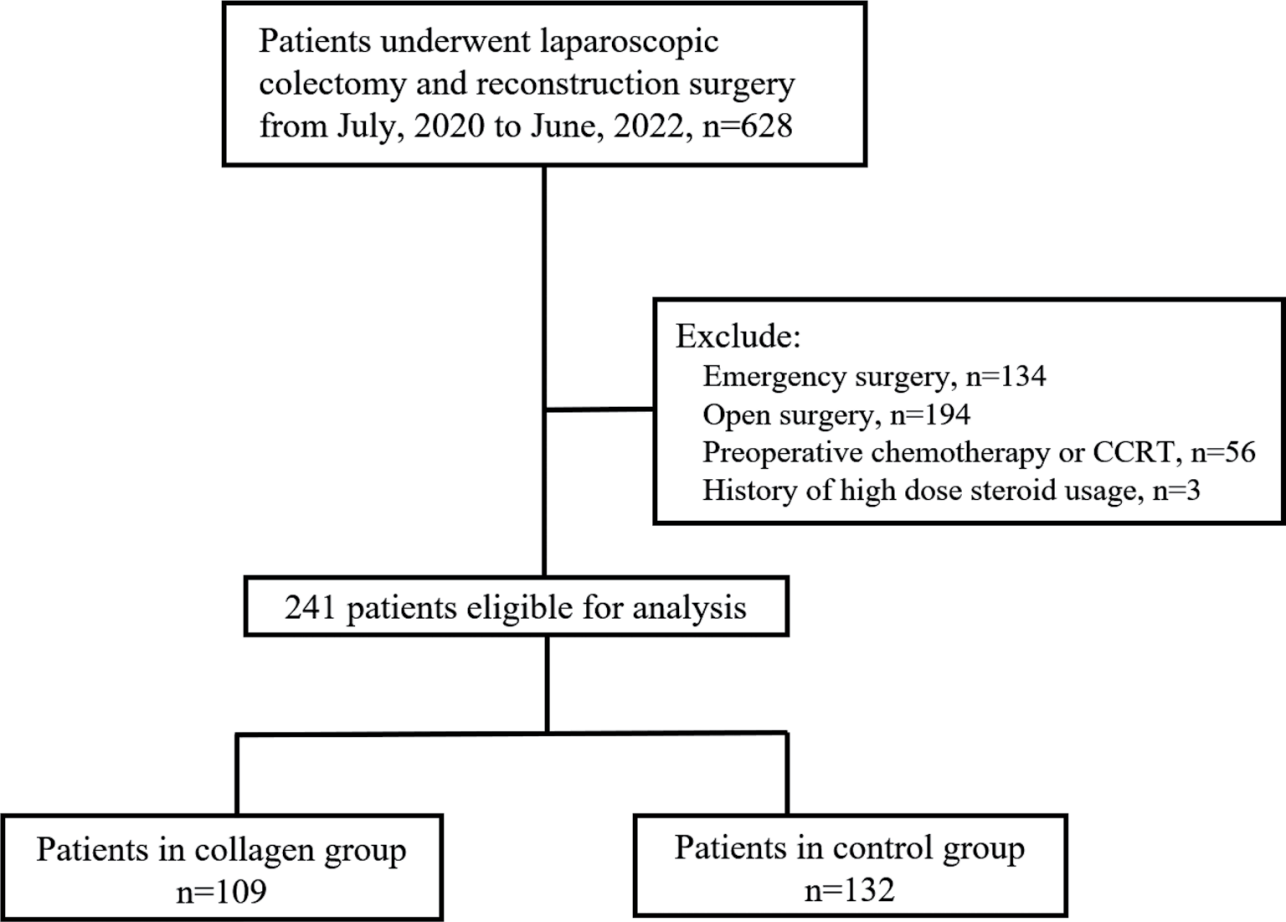
Flow diagram of selection of patients who underwent colorectal surgery in this study. The flowchart indicates the number of patients identified at each step and the reasons for exclusion. *Abbreviations* n, number; CPC, collagen patch cover; CCRT, concurrent chemoradiotherapy

A retrospective analysis was performed on the medical records of 628 consecutive patients who underwent primary colorectal resection and anastomosis at a single institute between July 2020 and June 2022, either for colorectal cancer or benign lesions (see Fig. 1). All patients receiving treatment during this specified period were included in the study. Patients who met the following criteria were excluded from the study: those who underwent open surgery, emergent surgery, an ostomy, or had a history of prior chemotherapy or concurrent chemoradiotherapy. These exclusion criteria were applied because these factors can considerably influence the degree of recovery. The following patient information was collected: age, sex, body mass index (BMI), Charlson Comorbidity Index (CCI) [16], clinical history, tumor location, stage of presentation, type and duration of surgery, postoperative morbidity, postoperative hospital stay, time to first flatus (TFF), time to first oral intake (TFO), time to first defecation (TFD) after surgery, and amount of drainage. TFF, TFO, and TFD are defined as follows: TFF is the number of days from operation to the first flatus, TFO is the number of days from operation to the first meal, and TFD is the number of days from operation to the first defecation.

### Surgical method of CPC application

After the anastomosis is completed, the abdominal cavity will be irrigated with normal saline. Once the irrigation is finished, any irrigated water inside the abdominal cavity will be suctioned out as much as possible. Subsequently, the anastomotic site will be thoroughly dried using gauze. Next, one 5 × 5 × 0.3 cm CPC (HealiAid, collagen wound dressing, Maxigan Biotech Inc.) will be halved to create two pieces of 2.5 × 5 × 0.3 cm Collagen patches. Two cut collagen patches will be applied to the anterior and posterior sides of the anastomotic site. Finally, a Jackson-Pratt drainage tube was placed at the anastomotic sites in each patient. The Jackson-Pratt drainage tube was employed in both groups of patients as a standard practice for colorectal resection at our institute. Patients who receive CPC application on the anastomotic site will be referred to as the collagen group, while patients without CPC application on the anastomotic site will be the control group.

### Outcomes

The primary outcomes were the number of days until the first flatus after surgery and the number of days until defecation after surgery, which was recorded as the time from surgery to the first occurrence of each event. The secondary outcomes were postoperative pain scores, intravenous (IV) analgesic use, daily amounts of drainage, and overall complications. Pain scores were evaluated daily using a visual analog scale (VAS), with scores ranging from 0 (*no pain*) to 10 (*the worst pain that I have ever experienced*). The frequency of IV analgesic use, amount of drainage, amount of intra-operative bleeding, and adverse events were analyzed; this information was collected by a nurse practitioner. Furthermore, we recorded each patient with the highest postoperative body temperature (BT) (recorded using an ear thermometer) during the first five postoperative days. We compared the preoperative white blood cell (WBC) count on the day before surgery with the postoperative WBC count coming morning after surgery. Postoperative complications were defined as clinical signs within 30 days of surgery (e.g., infection, leakage, hemorrhage, and paralytic ileus) and were classified by physicians using the Clavien–Dindo classification system [17]. The 30-day readmission and mortality rates were analyzed. The outcomes and complications of the two groups were compared.

### Statistical analysis

Statistical analysis was conducted using IBM SPSS Statistics for Windows, Version 27.0 (IBM Corp., Armonk, NY, USA). Continuous variables are presented as the mean ± standard deviation. Categorical variables are presented as numbers and percentages. Comparisons of groups were performed using the Student’s *t* tests and chi-square test for continuous and categorical variables, respectively. Odds ratios were calculated based on the ratio of patients with a TFF of ≥ 3 days to patients with a TFF of < 3 days, and the results were used to estimate the effects of various factors on recovery speed. Univariate and multivariate regression analyses were performed to determine the odds ratios (ORs) and 95% confidence intervals (CIs) for several variables that could influence the outcomes. All statistical tests were two-tailed, and a *p* value of < 0.05 was regarded as statistically significant.

### Ethics approval

This study was conducted in accordance with the guidelines of the Declaration of Helsinki and data were reported following the recommendations of the STROCSS 2019 guideline [18]. All procedures involving participants was approved by the Taipei Medical University Joint Institutional Review Board and Ethics Committee (TMU-JIRB; approval number: N202209004). The requirement for informed consent was also waived by the Taipei Medical University Joint Institutional Review Board and Ethics Committee because of the retrospective design of the present study.

## Results

After reviewing the medical records of 628 patients and applying the inclusion and exclusion criteria, 241 patients were determined to be eligible for inclusion (Fig. 1). The data from 109 (45.2%) patients assigned to the collagen group and 132 (54.8%) patients assigned to the control group were compared. The baseline characteristics are presented in Table 1. Age, sex, BMI, CCI score, and history of abdominal surgery were not significantly different between the groups. The proportion of patients with a smoking history was higher in the collagen group than in the control group (17.4% vs. 8.3%, *p* = 0.045). The collagen group comprised 20 patients (18.3%) with benign lesions and 89 patients (81.7%) with malignant tumors. The control group comprised 33 patients (25%) with benign lesions and 99 patients (75%) with malignant tumors. Benign lesions in the two groups were mostly benign tumors (collagen vs. control, 65% vs. 54.5%, *p* = 0.215), followed by diverticulosis (collagen vs. control, 30% vs. 30.3%, *p* = 0.215). No significant difference was identified between the two groups with respect to the classification of benign lesions and malignant tumor staging. Additionally, the data of the two groups did not exhibit any significant differences with respect to tumor size, length of resection, surgical type, anastomosis technique, blood loss, and number of lymph nodes harvested. However, the surgical time was longer in the collagen group than in the control group (215 min vs. 183.9 min, *p* < 0.01).

**Table 1:** Baseline characteristic of all patients

Characteristic	Collagen group (*N* = 109)	Control group (*N* = 132)	p value
Age, y	65.9 ± 12	66.7 ± 13.5	0.62 †
Sex			0.48 ‡
Male	62 (56.9)	69 (52.3)	
Female	47 (43.1)	63 (47.7)	
BMI	25.2 ± 3.8	24.8 ± 4.8	0.43 †
History of smoking	19 (17.4)	11 (8.3)	**0.045 ‡**
Benign, n	20	33	0.22‡
Benign tumor	13 (65)	18 (54.5)	
Diverticulosis	6 (30)	10 (30.3)	
Others	1 (5)	5 (15.2)	
Malignancy, n	89	99	0.22‡
Stage 1	31 (35)	24 (24)	
Stage 2	19 (21)	26 (26)	
Stage 3	31 (35)	39 (40)	
Stage 4	8 (9)	10 (10)	
Tumor size, mm	35 ± 18.1	35.6 ± 20.4	0.82 †
CCI	4.9 (2.2)	4.8 (2.6)	0.66 †
History of abdominal OP	25 (22.9)	27 (20.5)	0.64 ‡
Combined surgery	6 (5.5)	8 (6.1)	0.85 ‡
Length of resection, mm	213.4 ± 129.4	211 ± 132.9	0.87 †
Lymph nodes harvested, n	19.3 ± 6.6	18.9 ± 8.1	0.72 †
OP time, min.	215 ± 54.2	183.9 ± 62.7	**< 0.01 †**
Blood loss, c.c.	18.5 ± 59.2	26 ± 90.1	0.57 †
Surgical type			0.07 ‡
Right hemicolectomy	36 (33.0)	47 (35.6)	
Left hemicolectomy	15 (13.8)	4 (3)	
Anterior resection	38 (34.9)	56 (42.4)	
Lower anterior resection	17 (15.6)	19 (14.4)	
Segmental resection	2 (1.8)	4 (3)	
Subtotal colectomy	1 (0.9)	2 (1.5)	
Anastomosis technique			0.45 ‡
Intracorporeal	72 (66)	81 (61.4)	
Extracorporeal	37 (33.9)	51 (38.6)	

Data presented as mean ± standard deviation or n (%)

*Abbreviation*: SD, standard deviation; n, number; BMI, body mass index; Stage: The TNM staging system (AJCC Cancer Staging Manual, 8th Edition) was used for staging; CCI, Charlson Comorbidity Index; OP, operation; †, Student’s t test; ‡, chi-square test

Boldface indicates statistical significance (P **<** 0.05)

**Fig. 2:**
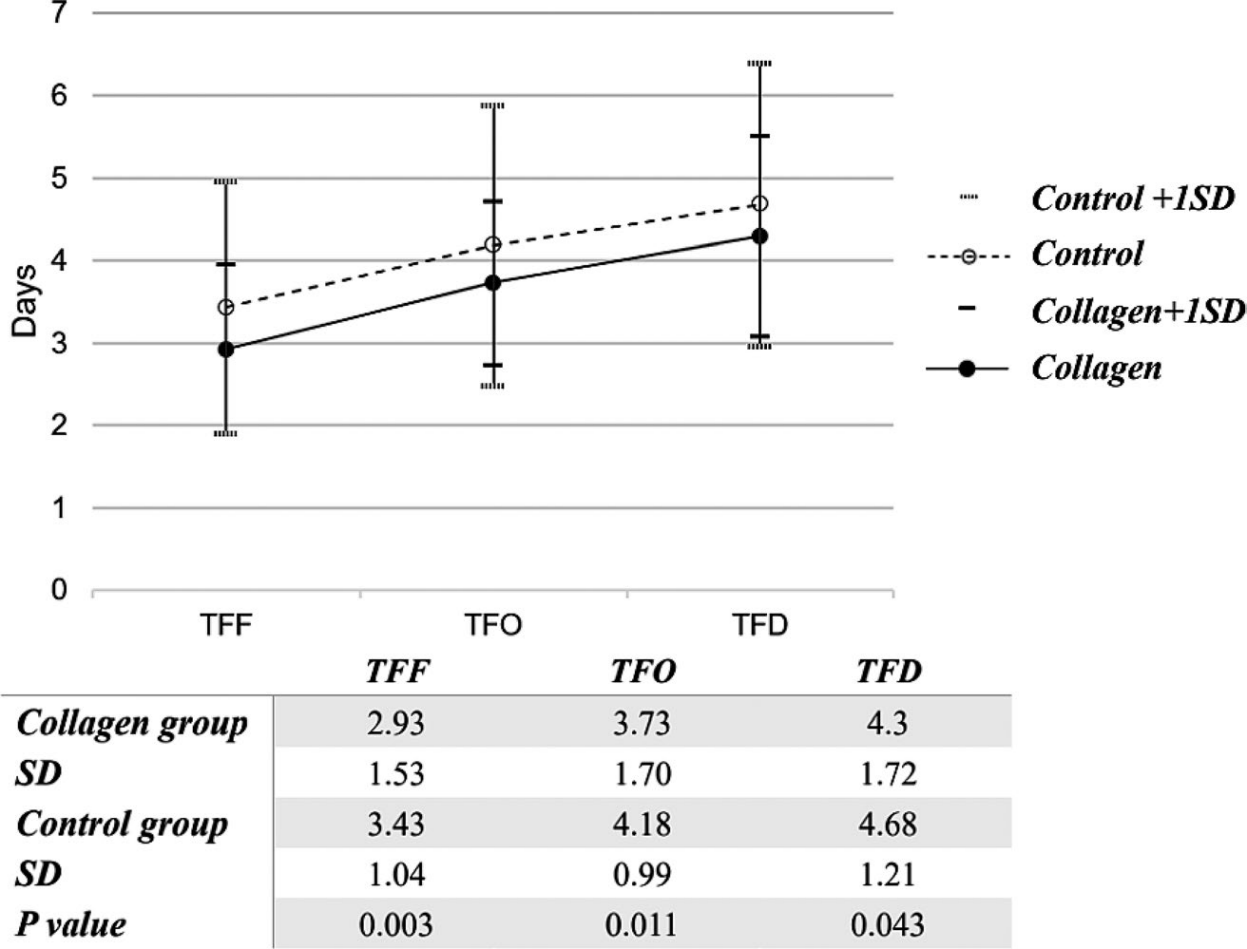
Analysis of the differences between the collagen and control group. *Y*-axis indicates the number of days from surgery to the first occurrence of an event (expressed as means and standard deviations). *X*-axis indicates event type. *Abbreviations* TFF, time to first flatus; TFO, time to first oral intake; TFD, time to first defecation

The postoperative outcomes of the two groups are compared in Table 2. The mean total IV analgesic dosage was lower in the collagen group than in the control group (collagen vs. control, 3.4 doses vs. 4.8 doses, *p* = 0.02). The two groups did not differ significantly in terms of mean daily pain scores from the first to the fourth day, daily amounts of drainage from the first to the sixth day, total amount of drainage, and length of postoperative hospital stay. The complication rates were not significantly different between the collagen group and the control group (17.4% vs. 13.6%, *p* = 0.42). The two groups did not differ significantly in their rates of developing paralytic ileus, infection, hemorrhage, and anastomotic leakage or in their degrees of complication, as determined using the Clavien–Dindo classification system. The proportion of patients with the highest BT ≥ 38 °C was not significantly different between the two groups. WBCs on the day before surgery and on the day after surgery were analyzed. The proportion of patients with a postoperative WBC count > 10,000/µL was significantly higher in the control group than in the collagen group (control vs. collagen, 68% vs. 55%, *p* = 0.036). The mean TFF, mean TFO, and mean TFD of the collagen group were significantly shorter than those of the control group (mean TFF, collagen vs. control, 2.93 days vs. 3.43 days, *p* < 0.01; mean TFO, collagen vs. control, 3.73 days vs. 4.18 days, *p* = 0.01; mean TFD, collagen vs. control, 4.30 days vs. 4.68 days, *p* = 0.04). The TFF, TFO, and TFD results are shown in Fig. 2.

**Table 2:** Postoperative outcomes of all patients and complications

Postoperative outcomes	Collagen group (*n* = 109)	Control group (*n* = 132)	p value
Number of days until first flatus	2.9 ± 1	3.4 ± 1.5	**< 0.01 †**
Number of days until first oral intake	3.7 ± 1	4.2 ± 1.7	**0.01 †**
Number of days until first defecation	4.3 ± 1.2	4.7 ± 1.7	**0.04 †**
VAS			
Day 0	2.5 ± 0.7	2.4 ± 0.9	0.29 †
Day 1	2 ± 0.7	1.9 ± 0.6	0.3 †
Day 2	1.6 ± 0.6	1.7 ± 0.6	0.35 †
Day 3	1.3 ± 0.6	1.5 ± 0.6	0.17 †
Day 4	1.3 ± 0.6	1.3 ± 0.6	0.96 †
Number of days of postoperative stay	9.7 ± 3.7	10.5 ± 6.1	0.26 †
Total amount of drainage, ml	1095 ± 1519.3	1215.5 ± 1305	0.51 †
Complication rate	19 (17.4)	18 (13.6)	0.42 ‡
Paralytic ileus	10 (9.2)	10 (7.6)	0.64 ‡
Surgical site infection	6 (5.5)	6 (4.5)	0.72 ‡
Hemorrhage	1 (1)	4 (3.7)	0.26 ‡
Anastomotic leakage	1 (1)	0 (0)	0.27 ‡
Complication severity ^a^			0.99 ‡
Grade 1	2 (10.5)	2 (11.1)	
Grade 2	13 (68.4)	12 (66.7)	
≥ Grade 3	4 (21.1)	4 (22.2)	
BT			0.489‡
≥ 38 °C	35 (32)	48 (36)	
< 38 °C	74 (68)	84 (64)	
WBC			**0.036‡**
> 10,000/µL	60 (55)	90 (68)	
≤ 10,000/µL	49 (45)	42 (32)	
Dosage of IV analgesic	3.4 ± 3	4.8 ± 5.8	**0.02 †**
Types of analgesic			0.12
No analgesic	12 (11)	21 (15.9)	
NSAID	34 (31.2)	24 (18.2)	
Opioids	45 (41.3)	62 (47)	
NSAID + Opioids	18 (16.5)	25 (18.9)	

Data presented as mean ± standard deviation or n (%)

*Abbreviations* d, day(s); SD, standard deviation; VAS, visual analog scale of pain; n, number; µL, microliter; IV, intravenous injection; NSAID, non-steroidal anti-inflammatory drugs; †, Student’s *t* test; ‡, chi-square test

Boldface indicates statistical significance (P < 0.05)

^a^ Complication severity was estimated by Clavien–Dindo Classification

Table 3 presents the factors affecting the shorter time to first flatus less than 3 days (early bowel recovery). Univariate and multivariate analysis were conducted for the analysis. A higher odds ratio was considered to indicate a slower recovery. Univariate analysis revealed that the recovery in the control group (OR = 2.12, CI: 1.21–3.72, *p* < 0.01) was significantly slower than that in the collagen group. The other factors that were associated with a slower recovery were a higher complication rate (OR = 1.90, CI:0.92–3.93, *p* < 0.01), the use of IV analgesic for > 3 dosages (OR = 2.29, CI:1.33–3.97, *p* < 0.01), having undergone right-sided colectomy surgery (OR = 2.10, CI:0.99–4.41, *p* = 0.05), and having undergone colectomy combined with other abdominal surgeries (OR = 4.01, CI:1.30–12.41, *p* = 0.01).

**Table 3:** Factors affecting the shorter time to first flatus (early bowel recovery)

Characteristic	Unadjusted analysis		Adjusted analysis	
	Odd ratio (95% CI)	p value	Odd ratio (95% CI)	p value
Groups				
Collagen	1		1	
Control	2.12 (1.21–3.72)	**< 0.01** ^a^	2.02 (1.12–3.67)	**0.02**
Sex				
Male	1			
Female	1.06 (0.62–1.82)	0.83		
Age				
≥ 65	1.36 (0.78–2.35)	0.28		
< 65	1			
BMI				
≥ 25	1.15 (0.67–1.97)	0.61		
< 25	1			
Smoking				
Yes	0.47 (0.18–1.20)	0.11^a^	0.52 (0.19 − 1.39)	0.19
No	1		1	
Comorbidity				
Yes	0.92 (0.53 − 1.59)	0.76		
No	1			
CCI > 5	1.60 (0.93 − 2.77)	0.09^a^	1.51 (0.84 − 2.70)	0.17
CCI ≤ 5	1		1	
History of abdominal surgery				
Yes	0.99 (0.51–1.90)	0.97		
No	1			
Tumor				
Benign	1			
Malignant	0.85 (0.45–1.60)	0.61		
Location				
Left	1		1	
Right	2.10 (0.99–4.41)	**0.05** ^a^	1.95 (0.91–4.15)	0.08
Surgery				
Combined surgery	4.01 (1.30–12.41)	**0.01** ^a^	3.83 (1.18–12.46)	**0.03**
Isolated surgery	1		1	
Complication				
Yes	1.90 (0.92–3.93)	**< 0.01** ^a^	1.53 (0.69–3.37)	0.29
No	1		1	
Dosage of using IV analgesic				
Dosage > 3	2.29 (1.33–3.97)	**< 0.01** ^a^	1.92 (1.07–3.42)	**0.03**
Dosage ≤ 3	1		1	

Odds ratios (95% CI) were calculated using the ratio of patients with a time to first flatus of ≥ 3 days to those with a time to first flatus of < 3 days. The significant variables were collagen, location, combined surgery, complications, and the number of doses of analgesic used

*Abbreviations* 95% CI, 95% confidence interval; BMI, body mass index; CCI, Charlson Comorbidity Index; IV, intravenous injection

Boldface indicates statistical significance (P **<** 0.05)

^a^ Only variables with a *p* value < 0.2 in the univariate analysis were included in the multivariate analysis

In multivariate analysis, the recovery of the control group was significantly slower (OR = 2.02, CI: 1.12–3.67, *p* = 0.02) than that of the collagen group. The other factors that prolonged recovery time were having undergone colectomy combined with other abdominal surgeries (OR = 3.83, CI: 1.18–12.46, *p* = 0.03) and the use of IV analgesics for > 3 dosages (OR = 1.92, CI: 1.07–3.42, *p* = 0.03).

## Discussion

To the best of our knowledge, this is the first comparative study on the effects of CPC use in human colorectal surgery. Studies that have investigated collagen use in the context of human gastrointestinal surgery have been limited by their small sample sizes and lack of comparisons [14, 15]. Parker conducted a non-randomized study and concluded that collagen wound dressings are safe and can be easily applied in human colorectal surgery [14]. Marano compared the complication rates of two cohorts and reported that collagen use reduced the complication rate in patients who underwent upper gastrointestinal surgery [15]. We collected clinical data of 241 patients who underwent elective laparoscopic colorectal surgery with colon anastomosis. In this study, patients in the collagen group had significantly shorter TFF, TFD, and TFO after surgery than those in the control group, indicating that they experienced faster bowel movement recovery than those in the control group after surgery. Furthermore, the present study revealed that patients in the collagen group required fewer administrations of IV analgesics than those in the control group. However, the effects of CPC in colorectal surgery on pain control remain unclear. A possible explanation is that CPC use may affect pain control by reducing inflammatory reactions, as evidenced by the higher proportion of patients in the control group having a postoperative WBC count > 10,000/µL than those in the collagen group.

Several intraoperative strategies can accelerate recovery from colorectal surgery. Intraoperative strategies to do so include ensuring that surgery is minimally invasive and ensuring normothermia and fluid normovolemia [19]. Collagen is a novel material that can be used for colonic anastomosis healing [20]. Pommergaard et al. [10] reported that the collagen matrix patch TachoSil reduced the colonic anastomotic leakage rate in a mouse model. Another study employed a porcine model to compare four sealant products and revealed that all products enabled the gallbladders of the experimental group to tolerate higher levels of pressure relative to those of the control group [21]. Pantelis et al. reported that CPC use can reduce anastomotic bursting pressure and improve the healing process in high-risk mice models [9]. Several studies have demonstrated that collagen covers can be safely used in a practical setting including small bowel and colon anastomosis sites [9, 11–13]. In the present study, the leakage rates in the two groups did not differ significantly. The healing process and bowel function recovery were clinically assessed based on TFF, TFD, and TFO, and they were all significantly shorter in the collagen group than in the control group.

Although CPC offers several benefits for colon anastomosis healing, it may have some shortcomings. In the present study, compared to the control group, the collagen group had significantly longer operation times, which could increase the risk of complications [22, 23]. These longer times may occur because of the time required for CPC application and because the anastomotic site must be dried before CPC can be applied. Additionally, variations in surgical procedures and individual surgeons may impact the overall surgical duration. In 2011, Schreinemacher et al. used a rat model and reported a higher bowel obstruction rate following CPC administration [24]. Ozel et al. noted that CPC may trigger inflammatory reactions in the experimental study evaluating the effect of collagen patch in colonic anastomotic healing [25]. In the present study, no significant differences were identified between the two groups with respect to anastomotic leakage, infection, paralytic ileus, hemorrhage, and overall complication rates. This finding indicates that CPC use does not increase the rate of complications in colorectal surgery.

Anastomotic leakage is a severe complication in colorectal surgery, and researchers have used animal models to study the application of collagen wound dressings as a novel biomaterial for maintaining anastomotic stability and reducing colon pressure [26, 27].

The specific mechanisms by which collagen influence local and systemic cellular or biochemical processes in human colorectal anastomosis are still not fully understood. A plausible explanation could be attributed to the significance of collagen as a fundamental component of the extracellular matrix. Collagen serves some purposes in the context of anastomosis. Firstly, it acts as a crucial structural support within the extracellular matrix [7]. Secondly, collagen plays a pivotal role in fostering the migration and proliferation of fibroblasts, which are vital for synthesizing new collagen fibers and facilitating the repair of damaged tissue [8]. Additionally, collagen has been linked to the promotion of angiogenesis, the process of forming new blood vessels. This augmentation of blood supply to the affected area is instrumental in supporting the delivery of essential nutrients and oxygen required for the healing process [8]. In summary, the utilization of collagen during colorectal surgery has been associated with a noticeable increase in vascular proliferation and fibroblastic activity. Studies conducted in a high-risk mice model have demonstrated a substantial enhancement in vascular proliferation and fibroblastic activity following the application of collagen during colorectal surgery [28]. Ongoing research is dedicated to investigating the use of collagen to improve bowel function recovery after colorectal resection and anastomosis. Pawlus et al. applied a collagen matrix to high-risk patients following D2 gastrectomy with Roux-en-Y anastomosis and reported an absence of leakage in six patients [29]. Other studies have focused on the benefits and mechanisms of collagen in wound healing [25–27]. The present study may be the first to discuss the potential benefits of CPC use in improving bowel movement and recovery.

The use of CPCs in surgical procedures can result in WBC changes and lower BT [30]. These changes may reduce the postoperative risk of inflammatory response and promote faster recovery. We applied cutoff thresholds of 10,000/µL for WBC and 38 °C for BT to distinguish normal and elevated WBCs and BTs. These thresholds are commonly used in clinical practice to identify patients who may experience an infection or inflammation. In the present study, the collagen group had a lower proportion of patients with a WBC count of > 10,000/µL than the control group. In other studies, CPC has been demonstrated to exhibit anti-inflammatory effects, which can promote the growth of new tissues and reduce inflammation [8]. Testing for C-reactive protein and interleukin-6 levels, which are primary markers of inflammation, is not routine test in patients who undergo colorectal surgery in our institute. We exam these markers only when the patient had symptoms or signs of infection. However, recent studies pointed out that C-reactive protein and interleukin-6 levels may serves as a well predictor for the anastomotic leakage and acute abdomen after colorectal surgery [31, 32]. Thus, the direct effects of CPC on inflammation could not be verified in the present study, and further research is required to clarify the role of CPC in reducing inflammation and improving bowel movement.

The current study has several limitations. Firstly, its retrospective design may introduce bias into the results. To mitigate bias, we excluded patients who underwent conventional open surgery, focusing on laparoscopic procedures known for better recovery [33]. Second, CPC use wasn’t covered by the National Health Insurance system, potentially causing a selection bias in the collagen group due to a smaller pool of eligible individuals. Third, surgeries were performed by different surgeons, introducing variability in experience and practices that could impact recovery outcomes. Fourth, our study found a strong correlation between collagen use and improved gastrointestinal function recovery. However, the lower use of analgesics in this group may contribute to this correlation. Additionally, recovery after surgery is influenced by various factors like anesthetics, surgical techniques, and varied NSAID or opioids usage, which were not fully addressed in this study. Finally, the follow-up duration was insufficient to determine the long-term effects of CPC use on bowel function. Thus, further research is needed to clarify these effects.

## Conclusion

CPC is a novel biomaterial for improving healing. The present study revealed that the application of CPC to anastomotic sites can accelerate bowel movement recovery in patients undergoing elective laparoscopic colectomy. CPC use is also associated with a reduced use of intravenous analgesics. Several studies have demonstrated that collagen can facilitate anastomotic healing and is safe to use in animal models. These results are broadly consistent with those of this study. The complication rates in the collagen and control groups in the present study were not significantly different. Future studies should focus on how CPC use affects biochemical parameters of inflammation and long-term clinical outcomes.

## Data Availability

The data supporting the findings of this study are available from the corresponding author, Tung-Cheng Chang, upon reasonable request.

## Declarations

### Statistical assistance

The authors wish to thank Li-Ying Chen for statistical assistance with this manuscript.
